# Feature selection for gene prediction in metagenomic fragments

**DOI:** 10.1186/s13040-018-0170-z

**Published:** 2018-06-07

**Authors:** Amani Al-Ajlan, Achraf El Allali

**Affiliations:** College of Computer and Information Sciences, Computer Science Department, King Saud University, Riyadh, Saudi Arabia

**Keywords:** Feature selection, mRMR, Gene prediction, Metagenomics, ORF, Prokaryotes

## Abstract

**Background:**

Computational approaches, specifically machine-learning techniques, play an important role in many metagenomic analysis algorithms, such as gene prediction. Due to the large feature space, current de novo gene prediction algorithms use different combinations of classification algorithms to distinguish between coding and non-coding sequences.

**Results:**

In this study, we apply a filter method to select relevant features from a large set of known features instead of combining them using linear classifiers or ignoring their individual coding potential. We use minimum redundancy maximum relevance (mRMR) to select the most relevant features. Support vector machines (SVM) are trained using these features, and the classification score is transformed into the posterior probability of the coding class. A greedy algorithm uses the probability of overlapped candidate genes to select the final genes. Instead of using one model for all sequences, we train an ensemble of SVM models on mutually exclusive datasets based on GC content and use the appropriated model to classify candidate genes based on their read’s GC content.

**Conclusion:**

Our proposed algorithm achieves an improvement over some existing algorithms. mRMR produces promising results in gene prediction. It improves classification performance and feature interpretation. Our research serves as a basis for future studies on feature selection for gene prediction.

## Background

Metagenomics is the study of genetic information in uncultured organisms obtained directly from the environment [1–3]. The term metagenomics was coined in 1998 by Handelsman et al. as, the total genetic information of microbiota found in an environmental sample [4, 5]. Studies have shown that the number of species present in a metagenome can reach thousands of different species [1]. Metagenomics analysis rely on different analysis pipelines in order to answer many questions such as identifying the organisms present in a given sample and what are these organisms doing.

Gene prediction is a fundamental step in most metagenomics analysis pipelines [6]. Gene prediction is the process of locating genes in genomic sequences [6, 7]. Initially, studies identified genes through reliable experiments on living cells and organisms. However, it is usually an expensive and time-consuming task [8]. Computational approaches are the most commonly used method for finding genes as they have proven their effectiveness in identifying genes in both genomes and metagenomes at a fraction of the cost and time of experimental approaches. Computational approaches are divided into similarity-based and content-based approaches [9, 10]. Similarity-based methods identify genes by searching for similar existing sequences. Basic Local Alignment Search Tool (BLAST) [11] is used to search for similarities between a candidate gene and existing known genes. However, this approach is expensive and cannot be used to discover novel genes or species. Content-based methods try to overcome these limitations using statistical approaches to detect variations between coding and non-coding regions [1, 8]. While, these approaches are very successful in genomic sequences, there is still work to be done for metagenomics due to the nature of the data [6, 12]. The greatest challenges for gene prediction algorithms in metagenomics are the short read-length and the incomplete and fragmented nature of the data [1, 13].

Machine learning is widely and successfully used in metagenomics analysis [14]. Various methods for predicting genes based on machine learning algorithms were developed. Orphelia [15], Metagenomic Gene Caller (MGC) [16], and MetaGUN [17] are examples of such tools. Orphelia is a web-based program designed to predict genes in short DNA sequences that have unknown phylogenetic origins. First, Orphelia extracts all open reading frames (ORFs) from input DNA sequences. Then, all ORFs are scored using the Orphelia gene prediction model, which consists of a two-stage machine learning approach. In the first stage, some features from the ORF are extracted using monocodon usage, dicodon usage and translation initiation sites [15]. Then, linear discriminants are employed to reduce the feature space. In the second stage, a neural network is used to combine the features from the previous step with the ORF length and GC content of the read. The neural network approximates the probability that an ORF encodes a protein. Finally, a greedy method is used to select the most likely genes from the ORFs that overlap by at most 60 bases [15]. MGC [16] is an improvement over Orphelia. The MGC algorithm uses the same two-stage machine learning approach but creates separate classification models based on several pre-defined GC-content ranges. It uses the appropriate model to predict genes in a fragment based on its GC content. Moreover, MGC uses two new features based on amino-acid usage in order to improve the overall gene prediction accuracy [16]. The MGC method shows that the use of separate learning models instead of a single model improves gene prediction performance. Both Orphelia [15] and MGC [16] use a linear discriminant classifier as a feature selection method that combines a large number of features to produce new features.

Feature selection can be considered as a preprocessing technique that aims to improve the performance of the classification, reduce training and build time and help to understand the domain [18–20]. Feature selection methods can be classified as wrapper, filter, embedded and hybrid methods according to the way that learning algorithms select features [20, 21]. Wrapping methods use supervised learning approaches to validate feature sets. Therefore, wrapper methods are computationally expensive and do not scale well to high-dimensional data [20, 22, 23]. In addition, search overhead, overfitting and low generality are other disadvantages of wrapper methods [20]. Filter methods use general characteristics of the dataset without the involvement of supervised learning algorithms [20, 23, 24]. Filter methods have more generality, require less computation and scale well to high-dimensional data [19, 20]. Hybrid methods combine filter and wrapper methods. For example, filter methods are used to select a specific number of features. Then, wrapper methods are applied to choose the final best features [23]. Filter methods is more suitable in our problem, because there are large number of features.

In this paper, we introduce a content-based approach that uses machine learning techniques to predict genes in metagenomic samples. We introduce a new method that use recent feature selection technique mRMR instead of combining features from single source into a new feature.

## Method

### Dataset

To evaluate the effectiveness of our approach, we use two datasets, including one for training and the other for testing the models. The training data were used by Orphelia [15] and the MGC [16]. The training data contain 7 million ORFs in 700 bp fragments that were excised from 131 fully sequenced prokaryotic genomes (bacterial and archaeal) [15] and their gene annotations that obtained from GenBank [25]. The 700 bp fragments are randomly excised to create 1-fold genome coverage from each training genome and 5-fold coverage for each genome in the testing dataset. Previous research prove the effectiveness of the use of separate learning model for several pre-defined GC ranges in increasing the prediction accuracy [16]. The training dataset was split into 10 mutually exclusive parts based on pre-defined GC ranges as shown in Table 1. Each GC range contains around 700,000 sequences (ORFs). We used 100,000 sequences for feature selection and 600,000 sequences were used to build the classification model. The testing data contain three archaeal genomes and eight bacterial genomes. Table 2 lists the genomes that were used in testing with the number of ORFs in each genome. ORFs are extracted from fragments and classified into coding and non-coding based on annotations in the genomes. We refer to both complete and incomplete ORFs simply as ORFs. Complete ORFs are sequences that contain both start and stop codons, while incomplete ORFs are either missing upstream codons, downstream codons or both [26].

**Table 1:** Training data

GC range	GC content ranges	Number of ORFs
1	0-36.57	713,474
2	36.57-41.57	716,896
3	41.57-46	728,133
4	46-50.14	705,792
5	50.14-54.28	741,691
6	54.28-58.14	710,639
7	58.14-61.85	705,692
8	61.85-65	724,478
9	65-68.28	729,822
10	68.28-100	742,300

**Table 2:** Testing data

Genomes	Gene bank accession no.	Number of ORFs
Archaeoglobus fulgidus	NC_000917	206,257
Methanocaldococcus jannaschii	NC_000909	111,202
Natronomonas pharaonis	NC_007426	241,784
Buchnera aphidicola	NC_002528	38,541
Corynebacterium jeikeium	NC_007164	239,797
Chlorobaculum tepidum	NC_002932	206,807
Helicobacter pylori	NC_000921	120,138
Prochlorococcus marinus	NC_007577	117,755
Wolbachia endosymbiont	NC_006833	86,338
Burkholderia pseudomallei	NC_006350	311,856
Pseudomonas aeruginosa	NC_002516	494,924

### The proposed method

**Fig. 1:**
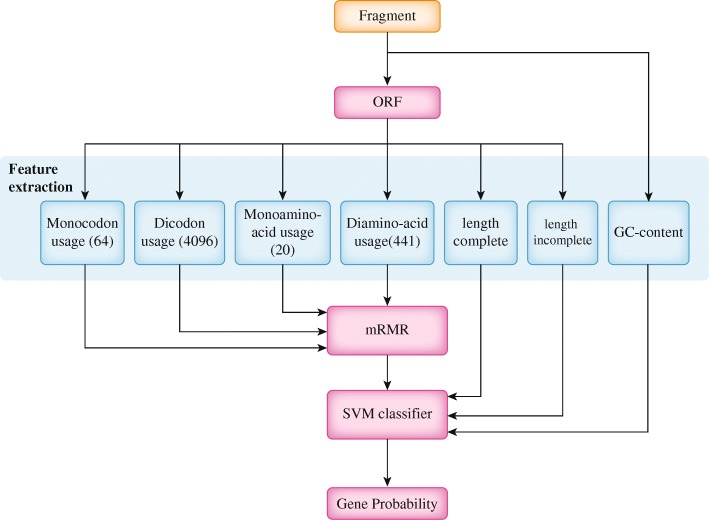
The proposed algorithm

Our proposed method consists of four phases: feature extraction, feature selection, training, classification and a post processing phase, as shown in Fig. 1. First, we extract a large number of features from each candidate ORF. Using mRMR, we select the effective and relevant features which are then used by SVM classifier to approximate the posterior probability of each ORF coding for gene. Finally, we use a greedy approach to select the final gene list.

#### Features extraction

In order to distinguish coding from non-coding sequences, we extract commonly used features in gene prediction: mainly codon and amino acid usages [7, 15, 16]. In addition to combining these usages into small set of features, gene finders also use features related to the translation initiation sites (TIS) such as the position weight matrices (PWM) around candidate sites. However, since not all our candidate ORFs are complete, we will not extract any TIS related features but rather rely on post-processing techniques to correct the TIS in our predictions [27]. The following shows the different categorizations of our features:

**monocodon usage:** The frequency of occurrences of each codon. Since there are 64 different codons, the monocodon usage produces 64 features.

**dicodon usage:** The frequency of pairs of successive half-overlapping codons. Dicodon usage produces 4,096 features.

**monoamino acid usage:** The frequency of occurrences of each amino acid [16]. Since there are 20 amino acids, this usage produces 20 features.

**diamino acid usage:** The frequency of pairs of successive half-overlapping amino acids. Diamino acid usage produces 441 features.

In addition to features based on usage, we also consider the following three features:

**ORF length ratios:** The ratio between the length of the candidate ORF and its read length. Since we have complete and incomplete ORFs, we compute two features (complete length ratio and incomplete length ratio). If the candidate ORF is complete, then its incomplete length feature is set to zero and vice versa.

**GC content:** The percentage of cytosine and guanine in the read is assigned a feature for all candidate ORFs extracted from the particular read. Usually, coding regions have a higher GC content than non-coding regions [28].

#### Feature selection

Our method uses minimum redundancy maximum relevance (mRMR) [29] as it scales well to high-dimensional data and has promising results under different applications [29, 30]. In 2005, Peng et al. proposed the mRMR filter-method which aims to select features that are maximally dissimilar to each other but as similar as possible to the classification variable [30]. Since our data are continuous, we use the F-test as a relevance measure and the Pearson correlation among variables as a redundancy measure. The maximum relevance of feature set *S* for class *c* is defined by the average value of all F-test values between the individual feature *i* and the class *c* as follows:

1 $$ max V_{F}, V_{F}=\frac{1}{\left | S \right |} \sum_{{i \in S}} F(i,c)  $$

The minimum redundancy of all features in feature set *S* is defined by the average value of all Pearson correlations between the feature *i* and the feature *j* as follows:

2 $$ min W_{c}, W_{c}=\frac{1}{\left | S \right |^{2}} \sum_{{i, j \in S}} \left | c(i,j) \right |  $$

where *c*(*i,j*) represents the Pearson correlation coefficient. We use an F-test with a correlation quotient (FCQ) as the mRMR optimization condition that combines the two above criteria of maximal relevance and minimal redundancy as follows:

3 $$ max \left(\frac{V_{F}}{W_{c}}\right)  $$

We explore different mRMR feature sizes and compute k-nearest neighbor classification error rates for each feature set. Table 3 shows the error rate for different number of features. The results are based on sequences with GC content between 50.40 and 55.90. We select the top 500 features for the next phase of our algorithm since the classification gain from 300 to 500 features is not significant (less than.1%). For each GC range, we repeated the same experiment and selected the best 500 features to train SVM models.

**Table 3:** Classification error rates vs. number of features

mRMR Feature-set size	Error rate
60	0.0321
200	0.0264
250	0.0257
300	0.0253
350	0.0249
400	0.0246
450	0.0245
**500**	**0.0242**

#### Training models

We train support vector machines to produce the posterior probabilities of the coding class. The posterior probability *P*(*class*/*input*) is the probability of a *class* given evidence *input*. This technique was first introduced by Platt in 1999 [31]. Platt proposed a method to extract posterior probability from the SVM output. In binary classification with two classes +1 and -1, for input *x*, the posterior probability is calculated by the following formula:

4 $$ P(y=1/x)=\frac{1}{1+exp(Af(x)+B)}  $$

where *A* and *B* are two scalar parameters that are learned by the algorithm and function *f* is the output from the SVM. Moreover, Platt [31] suggests transforming the label y to target probabilities *t*_+_ for positive samples and *t*_−_ for negative samples:

5 $$ t_{+}=\frac{N_{+}+1}{N_{+}+2}  $$

6 $$ t_{-}=\frac{1}{N_{-}+2}  $$

where *N*_+_ and *N*_−_ are the number of positive and negative samples, respectively.

In order to train the SVM, we first partition the training data to 10 mutually exclusive partitions using pre-defined GC ranges. The GC ranges were decided by simply dividing the training data into 10 partitions. Previous research shows that using smaller ranges does not improve the prediction [16]. Thus, an ensemble of SVM models based on GC content is used for classification. Each SVM model will produce the probability that a given ORF is a gene, as shown in Fig. 1. Prior to training the SVM models we use a grid search approach in order to tune the parameters for the RBF kernel of the SVM classifier. Table 4 presents the best cost and gamma for each GC range. The best features from mRMR and the best parameters from the grid search are used to create the final SVM models.

**Table 4:** Best RBF parameters for each GC range

GC range	Best cost	Best gamma	Accuracy	Sensitivity	Specificity
1	100	1.5	97.89	94.03	98.53
2	100	1.5	98.37	95.68	98.75
3	100	1.5	98.40	95.66	98.74
4	100	2	98.28	94.67	98.71
5	100	2	98.22	94.80	98.61
6	100	2	98.05	94.05	98.49
7	100	2	98.30	94.93	98.71
8	100	1.5	98.70	96.51	98.99
9	100	2	98.95	97.09	99.22
10	100	2	99.08	97.66	99.31

#### Classification and post-processing

In this stage, all complete and incomplete ORFs are extracted from each input fragment. Based on the GC content of the fragment, appropriately 500 features are extracted from each ORF. These features are the same features that were used to build the model. Additionally, we extract three more features: GC content of the fragment, complete length, and incomplete length of the ORF. Then, the appropriate SVM model based on the GC content of the fragment is selected to score the ORF. The output from the SVM is the probability that a given ORF is a gene. We consider ORFs with probability greater than 0.5 as candidate genes. However, some of candidate genes can be overlapped and only one of them can be a gene. Genes in prokaryotes can maximally overlap by 45 bp [32]. Thus, a greedy algorithm [15, 16] is used as a post-processing step to solve the overlap between candidate genes and select the final gene list. The candidate gene with highest probability is more likely to be a gene. Algorithm 1 describes the final candidate selection where *g* is the final gene list for a particular fragment and *C* contains the candidate list. To allow for direct comparison with other algorithms, we set the maximum overlap *o*_*max*_ to be the minimum gene length which is 60 bp. The last step is to run the post-processing tool to correct the TIS, such as MetaTISA [27].


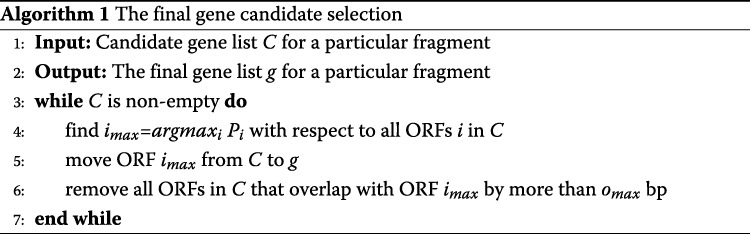


## Results and discussion

### Performance measures

Gene prediction performance is measured by comparing the model prediction with the true gene annotation in fragments that were obtained from GenBank [25]. Then, we count the number of true positives, false positives, and false negatives. True positive (TP) means the ORF correctly matched at least 60 bp in the same reading frame of annotated gene. False positive (FP) means the predicted ORF is incorrectly identified as a gene. False negative (FN) means an overlooked gene is incorrectly identified as non-coding. Then, we compute sensitivity, specificity, and harmonic means:

7 $$ Sensitivity=\frac{TP_{gene}}{TP_{gene}+FN_{gene}}  $$

8 $$ Specificity=\frac{TP_{gene}}{TP_{gene}+FP_{gene}}  $$

9 $$ Harmonic Mean=\frac{2 \times Sens \times Spec}{Sens + Spec}  $$

### Results

In this section, we compare the performance of the proposed mRMR-SVM algorithm with the mRMR-neural network algorithm for all 11 datasets presented in Table 2. Table 5 presents the comparison of the SVM and neural networks using the testing data. The results, as shown in Table 5, indicate that the SVM has a higher average harmonic mean than the neural networks: 92.17*%* and 90.33*%*, respectively. Based on these results, we select the mRMR-SVM algorithm for comparison with state-of-the-art algorithms.

**Table 5:** Comparison of SVM and neural network on testing data

	SVM			Neural network		
Genomes	Sp	Sn	H.M.	Sp	Sn	H.M
A. fulgidus	96.46	87.26	91.61	95.60	82.09	88.33
M. jannaschii	97.29	94.58	95.91	97.21	93.30	95.21
N. pharaonis	97.37	82.71	89.44	96.10	77.27	85.66
B. aphidicola	97.94	93.28	95.56	98.11	92.16	95.04
C. jeikeium	97.31	88.64	92.77	97.15	84.84	90.58
C. tepidum	95.93	80.84	87.74	94.71	75.99	84.32
H. pylori	97.67	92.09	94.80	97.45	91.28	94.26
P. marinus	98.58	87.65	92.79	98.71	85.22	91.47
W. endosymbiont	88.10	89.66	88.87	88.69	87.04	87.86
B. pseudomallei	97.56	85.83	91.32	97.95	81.43	88.93
P. aeruginosa	97.64	88.88	93.05	97.70	86.90	91.98
**Average**	**96.53**	**88.31**	**92.17**	**96.31**	**85.23**	**90.33**

**Fig. 2:**
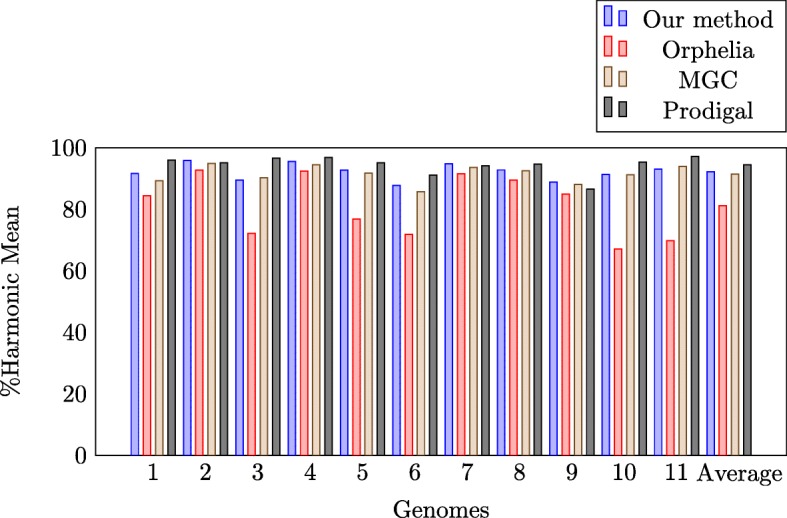
Harmonic mean of our method, Orphelia, MGC, and Prodigal

We compare our algorithm with state-of-the-art algorithms, namely, Orphelia [15], MGC [16], and Prodigal [33], using the testing data. We test 10 random replicas per genome and then, we compute the mean and standard deviation for the specificity, sensitivity, and harmonic mean for each genome. Table 6 and Fig. 2 present the comparison results. Our method achieves an average specificity of 96.53*%*, a sensitivity of 88.31*%*, and a harmonic mean of 92.17*%*. Our algorithm outperforms Prodigal in terms of specificity, but Prodigal outperforms our algorithm in terms of sensitivity and harmonic mean. In addition, our algorithm outperforms Orphelia and MGC in terms of specificity, sensitivity, and harmonic mean. Meanwhile, our method outperforms Orphelia by an average of 11% and MGC by an average of 1%. Our experiments confirm the MGC hypothesis that building several models based on several GC content is better than building a single model.

**Table 6:** Comparison of our method, orphelia, MGC and prodigal on testing data

	Our method (SVM)			Orphelia			MGC			Prodigal		
Genomes	Sp	Sn	H.M.	Sp	Sn	H.M	Sp	Sn	H.M	Sp	Sn	H.M
A. fulgidus	96.46 ±0.16	87.26 ±0.19	91.61 ±0.11	88.57 ±0.21	80.58 ±0.17	84.38 ±0.16	95.04 ±0.14	84.13 ±0.23	89.31 ±0.15	95.79 ±0.15	96.13 ±0.08	95.96 ±0.10
M. jannaschii	97.29 ±0.13	94.58 ±0.15	95.91 ±0.12	95.20 ±0.17	90.46 ±0.16	92.77 ±0.14	97.19 ±0.12	92.63 ±0.19	94.85 ±0.13	95.14 ±0.14	95.15 ±0.15	95.15 ±0.12
N. pharaonis	97.37 ±0.08	82.71 ±0.20	89.44 ±0.13	75.99 ±0.34	68.74 ±0.34	72.17 ±0.33	95.28 ±0.12	85.79 ±0.20	90.29 ±0.14	97.48 ±0.10	95.77 ±0.18	96.62 ±0.12
B. aphidicola	97.94 ±0.11	93.28 ±0.37	95.56 ±0.22	95.54 ±0.28	89.40 ±0.33	92.37 ±0.22	98.01 ±0.19	91.11 ±0.37	94.43 ±0.23	96.65 ±0.27	96.97 ±0.26	96.81 ±0.25
C. jeikeium	97.31 ±0.11	88.64 ±0.21	92.77 ±0.14	79.52 ±0.22	74.23 ±0.23	76.79 ±0.22	96.13 ±0.11	87.70 ±0.23	91.72 ±0.17	95.31 ±0.19	94.99 ±0.10	95.15 ±0.10
C. tepidum	95.93 ±0.12	80.84 ±0.23	87.74 ±0.17	77.51 ±0.22	66.95 ±0.23	71.85 ±0.21	93.42 ±0.14	79.08 ±0.24	85.65 ±0.18	94.35 ±0.14	88.15 ±0.19	91.14 ±0.11
H. pylori	97.67 ±0.12	92.09 ±0.21	94.80 ±0.12	94.17 ±0.20	88.99 ±0.22	91.5 ±0.20	97.77 ±0.14	89.70 ±0.22	93.56 ±0.17	95.29 ±0.14	93.07 ±0.14	94.16 ±0.12
P. marinus	98.58 ±0.07	87.65 ±0.25	92.79 ±0.16	94.41 ±0.20	84.984 ±0.24	89.45 ±0.20	97.71 ±0.11	87.92 ±0.20	92.55 ±0.12	97.52 ±0.17	91.96 ±0.20	94.66 ±0.15
W. endosymbiont	88.10 ±0.33	89.66 ±0.20	88.87 ±0.20	86.24 ±0.20	83.79 ±0.20	84.99 ±0.20	88.25 ±0.20	87.85 ±0.20	88.05 ±0.20	81.52 ±0.41	92.27 ±0.25	86.56 ±0.31
B. pseudomallei	97.56 ±0.06	85.83 ±0.19	91.32 ±0.11	69.54 ±0.31	64.79 ±0.22	67.08 ±0.26	94.79 ±0.13	87.84 ±0.25	91.18 ±0.18	94.28 ±0.09	96.47 ±0.09	95.37 ±0.08
P. aeruginosa	97.46 ±0.08	88.88 ±0.14	93.05 ±0.11	71.21 ±0.20	68.40 ±0.18	69.78 ±0.19	96.16 ±0.09	91.70 ±0.11	93.88 ±0.08	96.47 ±0.05	97.88 ±0.06	97.17 ±0.05
**Average**	**96.53**	**88.31**	**92.17**	**84.35**	**78.30**	**81.19**	**95.43**	**87.76**	**91.40**	**94.53**	**94.44**	**94.43**
**Average S.D.**	**0.12**	**0.21**	**0.15**	**0.25**	**0.24**	**0.22**	**0.15**	**0.22**	**0.16**	**0.17**	**0.15**	**0.14**

### Discussion

The aim of our study is to apply feature selection techniques to metagenomics gene prediction. The motivation for applying feature selection is to improve gene prediction, reduce computational time, and increase domain understanding. Overall, the results provide important insights into using feature selection techniques in gene prediction. The experiments show the power of the mRMR-SVM framework. Furthermore, our experiments show that only a small number of features among thousands contribute to accurate gene prediction. mRMR selects the top 500 features and creates a balance between prediction accuracy and computational cost. Our method outperforms Prodigal in terms of specificity, and the overall performance of our method is higher than some prominent gene prediction programs, such as Orphelia and MGC. Additionally, our method and MGC achieve better results than Orphelia because both methods use several pre-defined GC classification models instead of a single model. There are some differences between our method and MGC. First, MGC uses a linear discriminant classifier method. Our method uses mRMR, which selects the features that correlate the strongest with a classification variable and that are mutually different from one another. Second, our method uses the SVM classifier, while MGC uses neural networks. Third, MGC has a feature called the Translation Initiation Site (TIS) score. In our study, we pick the leftmost TIS of each ORF-set, because the next step is to use the MetaTISA program [27] to correct the TIS.

### Conclusion

We investigate the use of feature selection in gene prediction for metagenomics fragments. This is an important step toward enhancing the gene prediction process. We use filter feature selection methods because they scale well for high-dimensional data. We propose applying the mRMR algorithm to our data to reduce features and then apply the SVM to find the gene probability. Future work will investigate the use of deep learning to predict genes in metagenomics fragments. Deep learning is successfully used in bioinformatics and is able to handle a large number of features.
